# Efficacy of Food Supplement Based on Monacolins, γ-Oryzanol, and γ-Aminobutyric Acid in Mild Dyslipidemia: A Randomized, Double-Blind, Parallel-Armed, Placebo-Controlled Clinical Trial

**DOI:** 10.3390/nu16172983

**Published:** 2024-09-04

**Authors:** Lorenza Francesca De Lellis, Maria Vittoria Morone, Daniele Giuseppe Buccato, Marcello Cordara, Danaè S. Larsen, Hammad Ullah, Roberto Piccinocchi, Gaetano Piccinocchi, Paulraj Balaji, Alessandra Baldi, Alessandro Di Minno, Hesham R. El-Seedi, Roberto Sacchi, Maria Daglia

**Affiliations:** 1 Department of Pharmacy, University of Naples “Federico II”, Via D. Montesano 49, 80131 Naples, Italy; lo.delellis2@libero.it (L.F.D.L.); d.buccato@gmail.com (D.G.B.); hammadrph@gmail.com (H.U.); alessandra.baldi.alimenti@gmail.com (A.B.); maria.daglia@unina.it (M.D.); 2 Department of Experimental Medicine, Section of Microbiology and Clinical Microbiology, University of Campania “L. Vanvitelli”, 80138 Naples, Italy; mariavittoria.morone@unicampania.it; 3 School of Medicine, University of Milano-Bicocca, 20126 Milan, Italy; m.cordara@campus.unimib.it; 4 School of Chemical Sciences, The University of Auckland, Auckland 1010, New Zealand; d.larsen@auckland.ac.nz; 5 Level 1 Medical Director Anaesthesia and Resuscitation A. U. O. Luigi Vanvitelli, Via Santa Maria di Costantinopoli, 80138 Naples, Italy; roberto.piccinocchi@policliniconapoli.it; 6 Comegen S.c.S., Società Cooperativa Sociale di Medici di Medicina Generale, Viale Maria Bakunin 41, 80125 Naples, Italy; gpiccino@tin.it; 7 PG and Research Centre in Biotechnology, MGR College, Hosur 635130, TN, India; balaji_paulraj@yahoo.com; 8 CEINGE-Biotecnologie Avanzate, Via Gaetano Salvatore 486, 80145 Naples, Italy; 9 Department of Chemistry, Faculty of Science, Islamic University of Madinah, Madinah 42351, Saudi Arabia; hesham@kth.se; 10 Applied Statistic Unit, Department of Earth and Environmental Sciences, University of Pavia, Viale Taramelli 24, 27100 Pavia, Italy; roberto.sacchi@unipv.it; 11 International Research Center for Food Nutrition and Safety, Jiangsu University, Zhenjiang 212013, China

**Keywords:** mild dyslipidemia, red yeast rice, monacolin K, γ-aminobutyric acid, γ-oryzanol, randomized clinical trial

## Abstract

The risk of cardiovascular disease (CVD) is approximately doubled in subjects with hypercholesterolemia compared to those with normal blood cholesterol levels. Monacolin K (MK), the main active substance in rice fermented by the *Monascus purpureus*, acts on cholesterol metabolism. Rice also contains other bioactive compounds such as γ-oryzanol (OZ) and γ-aminobutyric acid (GABA). In a randomized, placebo-controlled, double-blind trial, the efficacy and tolerability of a food supplement (FS) based on an ingredient standardized to contain monacolins (4.5%), OZ, and GABA were evaluated in subjects with mild dyslipidemia. For the duration of the trial, enrolled subjects (*n* = 44, each group) received the FS or placebo and were instructed to use an isocaloric diet. Compared to the placebo group, after a 3 months of the FS, the mean low-density lipoprotein cholesterol and mean TC values were reduced by 19.3 and 8.3%, respectively, while the mean high-density lipoprotein cholesterol value increased by 29.3%. On average, the subjects shifted from very high to moderate CVD risk. Glucose metabolism and hepatic and renal parameters did not change after the treatment and no adverse events were reported. Guidelines to handle hypercholesterolemia with food supplements in specific clinical settings are needed to better manage mild dyslipidemia.

## 1. Introduction

Cardiovascular disease (CVD) is the main cause of mortality in developed countries [1]. Hypercholesterolemia has the highest pathophysiological and prognostic impact on atherosclerotic CVD. In fact, people with hypercholesterolemia have about double the risk of developing CVD than those with normal levels of total cholesterol [2]. In addition, patients with familial hypercholesterolemia (FH) have a very high risk of developing CVD at younger age. Thus, early diagnosis and treatment of hypercholesterolemia are critical to reduce CVD and premature death [3]. To keep cholesterol levels within the recommended levels and reduce the incidence of CVD [3], a healthy and balanced diet, combined with physical activity, and the use of statins when needed are the strategy of choice [3]. However, adverse events leading to intolerance and non-adherence are major determinants of poor therapeutic outcomes in statin therapy. Consumption of over-the-counter food supplements containing bioactive ingredients acting on different metabolic targets of cholesterol metabolism is considered an alternative strategy. Red yeast rice (RYR), a food ingredient used in traditional Chinese medicine, is a product of yeast (*Monascus purpureus*) cultivated on rice (*Oryza sativa* L.). Data from the literature support the cholesterol-lowering potential of RYR. Monacolin K (MK), which has the same chemical structure as lovastatin, is the main active substance acting on cholesterol metabolism present in RYR. A high-degree competitive inhibition of HMG-CoA (3-hydroxy-3-methylglutaryl-coenzyme A) reductase, a limiting step in cholesterol synthesis, is the mechanism of the lowering effect of MK on cholesterol levels [4]. With few exceptions, studies published on the effects of RYR support the clinical efficacy of this product in reducing hypercholesterolemia and sustaining normal circulating levels of plasma cholesterol in humans [5,6,7]. Besides MK, rice contains other bioactive substances, e.g., isovitexin, phytosterols, octacosanol, squalene, tocopherol and tocotrienol derivatives, γ-aminobutyric acid (GABA), γ-oryzanol (OZ). The latter is a mixture of plant sterols, triterpene alcohols, and ferulic acid esters that are found in grains (e.g., rice) [8]. By limiting exogenous cholesterol absorption, OZ exerts cholesterol-lowering activity, which supports its use in food supplements aimed at reducing high blood cholesterol levels. Pre-germinated brown rice rich in OZ and GABA significantly lowered blood cholesterol in hypercholesterolemic Sprague–Dawley male rats, as compared to non-germinated brown rice [9]. A more recent preclinical study showed that, compared to mice fed with MK alone, animals fed with the combination of MK, OZ, and GABA exhibited a greater lipid-lowering effect and normalization of inflammatory markers [10]. However, when tested versus MK in a randomized, placebo-controlled clinical trial, GABA-rich RYR (*Monascus pilosus*) did not produce significant lowering effects on serum lipid levels [11].

Considering that the information available does not predict higher/lower effectiveness in the use of the combination of MK, OZ, and GABA in humans, we evaluated the efficacy and tolerability of a food supplement based on an ingredient of natural origin standardized to contain monacolins (4.5%), γ-aminobutyric acid, and γ-oryzanol in subjects with mild dyslipidemia and without known cardiovascular disease.

## 2. Materials and Methods

### 2.1. Food Supplement and Placebo

The food supplement ‘ROSSOPURO^®^ Forte’ is investigated in this clinical trial, which is a proprietary ingredient of natural origin standardized to contain monacolins (4.5%), γ-oryzanol, and γ-aminobutyric acid (GABA) (Giellepi S.p.A., Milan, Italy). The European standards for microbiologic limits and contaminants were met by Giellepi S.p.A. (Milan, Italy), which supplied both verum and placebo at no cost. The Italian Health Ministry had previously been notified about the food supplement containing ROSSOPURO^®^ Forte (notification number: 159826).

According to European regulation [12], which states that a single serving of RYR-based food supplements for daily consumption should provide less than 3 mg of monacolins, each vegetable capsule of the investigational product (verum) contains 62.2 mg of ROSSOPURO^®^ Forte or 2.8 mg of MK. The vegetable capsule of placebo contains comparable amounts of maltodextrins.

### 2.2. Study Design

The effects of the ROSSOPURO^®^ Forte were assessed in otherwise healthy adults with a mildly impaired lipid metabolism in a monocentric, randomized, placebo-controlled, parallel-group, double-blind clinical trial conducted by COMEGEN—Società Cooperativa Sociale (Naples, Italy). The enrolled participants were randomized in two experimental groups (*n* = 44, each group). The dose forms were identical in terms of color, shape, weight, and flavor, and the packaging was designed to make it impossible to distinguish between the food supplement and the placebo. Before giving their written consent, participants were informed about this study both orally and in writing. The protocol, the participants’ letter of intent, and the study synopsis were approved by the Ethics Committee of A.S.L. Napoli 1 CENTRO (protocol number 294, 20 June 2023), and this study was conducted in accordance with the Helsinki Declaration of 1964 (as revised in 2000). This study is listed on the ISRCTN registry under the ID number ISRCTN90678255 [13]. To identify subjects who met the requirements to be enrolled in this study, total cholesterol (TC), low-density lipoprotein cholesterol (LDL-C), and high-density lipoprotein cholesterol (HDL-C) were determined during the screening visit (tr). The experimental groups included subjects who took a capsule of the food supplement based on ROSSOPURO^®^ Forte, containing MK, OZ, and GABA (GROUP 1), and subjects who took a placebo capsule (GROUP 2). After a 15-day run-in period, demographics, clinical history, and individual informed consent forms were collected at baseline, at the start of this study (i.e., at t0). At baseline (t0) and after 90 days (t1), the following biochemical parameters of the recruited subjects were determined after blood sampling: TC, LDL-C, HDL-C, triglycerides (TGs), fasting blood glucose (FBG), glycated hemoglobin A1c (HbA1c), erythrocyte sedimentation rate (ESR), C-reactive protein (CRP), white cell count, creatinine (CRE), alanine transaminase (ALT), and aspartate transaminase (AST). Moreover, body mass index (BMI) and abdominal circumference were determined at t0 and t1. This study took seven months to complete, two months for patient enrollment, one month for the run-in, and four months for the study, considering the timing for the last enrolled patient.

### 2.3. Participants and Recruiting

Based on the predetermined inclusion and exclusion criteria, 88 study participants of either sex (aged 18–70 years) were randomized into two groups. Inclusion criteria required that participants exhibited borderline TC values (i.e., 200–239 mg/dL) and LDL-C values ≤159 mg/dL. Individuals, identified as being at high risk of cardiovascular events, as determined by age, sex, smoking habit, diabetes, systolic blood pressure, TC, HDL-C, and antihypertensive treatment [14], were excluded from this study. Exclusion criteria also encompassed individuals receiving cholesterol-lowering drugs (even at low doses); those who had received supplements for cholesterol, blood sugar, and metabolic syndrome control in the two weeks prior to recruitment; women who were pregnant, currently lactating, suspected pregnant, or expected to become pregnant; and those who had donated blood in the three months prior to recruitment. Individuals who were not self-sufficient, those unwilling to cooperate, those unable to adhere to visit schedules, or who were deemed unsuitable by the investigator due to other diseases, were also not included. Enrolled subjects were instructed to use an isocaloric diet for the duration of this study. Such diet fostered the consumption of fruits, vegetables, carbohydrates from whole grains (fiber), low-fat milk derivatives, fish, white meat, and vegetable oils, and included a substantial decrease in intake of red meat, animal fats, salt, simple sugars, and alcohol. Dietary recommendations from the DASH (Dietary Approaches to Stop Hypertension) guidelines were given to participants. In addition to promoting blood pressure control, this eating pattern improves overweight/obesity control, insulin resistance, and hyperlipidemia [15,16]. With regard to macronutrient composition, the DASH eating pattern’s daily nutritional goals are as follows: total fat (27% of calories); saturated fat (6% of calories); protein (18% of calories); carbohydrates (55% of calories); cholesterol (150 mg); sodium (2300 mg); potassium (4700 mg); calcium (1250 mg); magnesium (500 mg); fiber (30 g). The assessment of diet compliance was performed using a food diary, which was provided to participants at t0 and collected at t1.

### 2.4. Study Outcomes 

Evaluating the effectiveness of the food supplement in lowering TC and LDL-C and improving lipid metabolism in participants with mildly impaired lipid levels was the primary outcome of the current investigation. Evaluating beneficial effects on glucose metabolism (FBG and HbC1A), BMI, and abdominal circumference reduction were secondary outcomes.

### 2.5. Safety and Tolerability

The food supplement under study contains ingredients that are all considered safe and are permitted by current European food legislation. Although no adverse events linked to the food supplement intake were foreseen, the subjects were continuously monitored as to suspected adverse reactions to be reported via the VigiErbe online phytovigilance system [17], according to the provisions of the Italian Istituto Superiore di Sanità. Suspected unexpected serious adverse reactions (SUSARs) during the trial would also have been reported to the Ethics Committee, which provided feedback on the clinical trial.

### 2.6. Statistical Analysis

Three 1-β power values (0.80, 0.95, and 0.99), two effect size values (Cohen’s f = 0.25 and 0.39, respectively), and a significance threshold value of α equal to 0.05 were used to calculate the sample size. The estimated size of the population sample for this clinical study was 84 subjects; an additional 5% of subjects were added to account for potential dropouts, for a total of 88 subjects enrolled (*n* = 44, each group). Linear mixed models (LMMs) were employed to analyze how study subjects responded to the experimental treatment. These models used the 12 response variables (FBG, HbA1c, TC, HDL-C, LDL-C, TG, ESR, CRP, white blood cell (WBC) count, ALT, AST, and CRE) as dependent variables, each in a separate model. For all models, the fixed effects included the measurement (t0 vs. t1) and the treatment (GROUP 1 vs. GROUP 2), along with their interaction. Additionally, we controlled for patient effects by adding sex, age, BMI, and waist circumference to the fixed part of the model. Age, BMI, and waist circumference were standardized (mean = 0 and SD = 1). Finally, we included patient identity as a random effect to account for within-subject repetition of measurements. The lme4 [18] package in R ver. 4.0.1 [19] was used to conduct the analyses. Unless otherwise indicated, data are presented as means and standard errors.

## 3. Results

**Figure 1:**
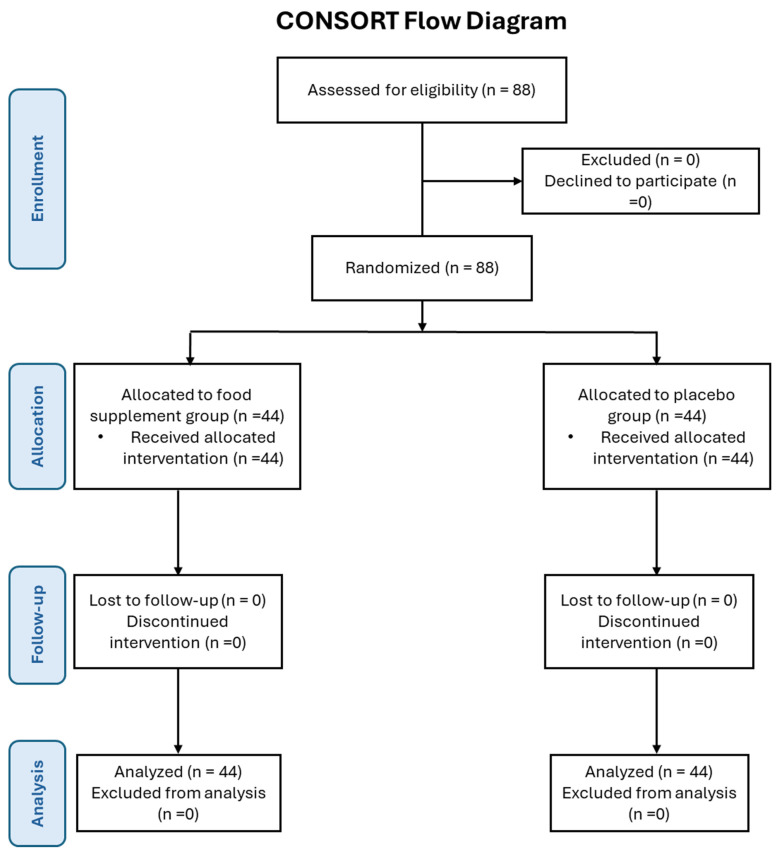
CONSORT flow diagram.

Figure 1 displays a study flowchart, produced following CONSORT PRO reporting guidelines [20]. Table 1 presents the baseline (t0) demographic and clinical data about the enrolled participants. The sample includes 44 subjects for each experimental group (26 women and 18 men for the placebo group, and 20 women and 24 men for the food supplement group). The mean age (±SD) of subjects was 57 ± 7 years and 53 ± 5 for the placebo group and food supplement group, respectively. Table 2 shows the descriptive statistics for the comparison between placebo group and food supplement group at t0 and t1 for each of the selected variables.

**Figure 2:**
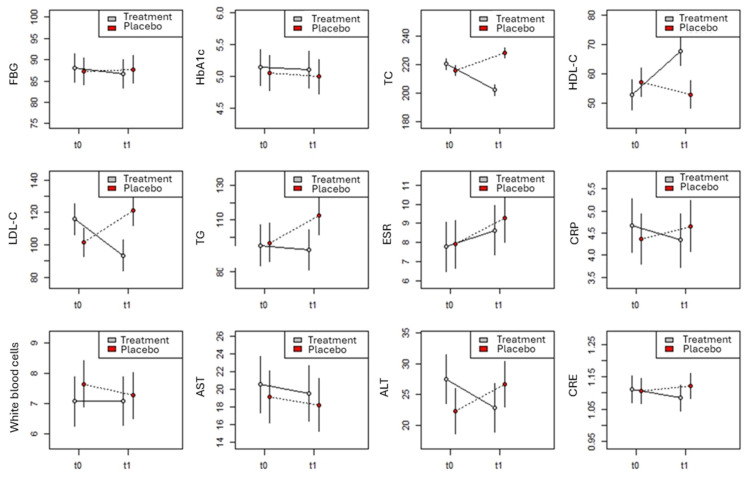
Comparison between TRT and PLA groups in the 12 variables measured in the trial as predicted by LMM models.

The findings of the LMM analysis are displayed in Table 3, Table 4 and Table 5 and Figure 2. A considerable effect was observed for the group and the interaction between group and measure in relation to TC, as indicated in Table 3. Notably, the effect of the subject’s sex was also significant. Specifically, TC values significantly increased in the placebo group (β = 12.4 ± 2.4, t88 = 5.264, *p* < 0.001, Figure 2), while they significantly decreased in the treated group (β = 18.3 ± 2.4, t86 = 7.820, *p* < 0.001, Figure 2). Although the effects of placebo and treatment were not statistically different from one another (β = 4.47 ± 2.4, t167 = 1.872, *p* = 0.06, Figure 1) at t0, the mean TC value of the placebo group was higher than that of the treated group at t1 measurement (β = 26.2 ± 2.4, t166 = 10.845, *p* < 0.001, Figure 2). Furthermore, as expected [21], regardless of treatment and measurement, men consistently exhibited higher TC values than women (β = 3.9 ± 1.7, t_82_ = 2.274, *p* = 0.026, Figure 2).

The model for the HDL-C value (Table 3) identified a significant effect for the measure, the group, and their interaction. In the placebo group, there was no significant change in the mean HDL-C value between t0 and t1 (β = 4.3 ± 3.1, t89 = 1.360, *p* = 0.18, Figure 2), while it increased significantly in the treated group (β = 15.1 ± 3.1, t87 = 4.789, *p* < 0.001, Figure 2). Consequently, while the placebo and treatment groups at t0 did not differ significantly (β = 4.4 ± 3.2, t167 = 1.390, *p* = 0.17, Figure 2), at t1, the mean HDL-C value of the treated group was significantly higher than that of the placebo group (β = 15.0 ± 3.2, t167 = 4.627, *p* < 0.001, Figure 2).

The model for LDL-C values (Table 3) identified a significant effect for the interaction between measurement and treatment alone. Specifically, the values in the placebo group increased significantly from t0 to t1 (β = 19.6 ± 5.7, t89 = 3.430, *p* < 0.001, Figure 2), while it decreased significantly (β = 22.6 ± 5.7, t87 = 3.996, *p* < 0.001, Figure 2) in the treated group. Consequently, while at t0, the treated group had a higher mean LDL-C value than that of the placebo group (β = 14.6 ± 6.0, t165 = 2.419, *p* = 0.017, Figure 2), the placebo group had higher mean LDL-C value than that of the treated group at t1 measurement (β = 27.6 ± 6.0, t_164_ = 4.534, *p* < 0.001, Figure 2).

The effect for TGs did not detect any significant effect (Table 3), even though it approached a statistical significance for both the group (*p* = 0.051) and the group × measure interaction (*p* = 0.08). When looking for model coefficients, the TG value did not vary between t0 and t1 in the treated group (β = 2.7 ± 7.4, t_168_ = 0.367, *p* = 0.71, Figure 2), but it increased in the placebo group (β = 15.8 ± 7.4, t168 = 2.143, *p* = 0.033, Figure 2). Consequently, while at t0, there were no significant differences between experimental groups (β = 1.3 ± 7.4, t_168_ = 0.180, *p* = 0.86, Figure 2), at t1, the mean TG value was higher in the placebo group than in the treated group (β = 19.9 ± 7.4, t_168_ = 2.638, *p* = 0.0091, Figure 2).

The LMM models for FBG and HbA1c (Table 4) did not reveal any significant effects. Consequently, the two variables remained unchanged in response to the treatment (Figure 2). No significant effects emerged for treatment, measurement, and their interaction in the models for ESR, CRP, WBC count, CRE, and AST (Table 4 and Table 5). The values of these variables remained substantially unchanged between t0 and t1 in both experimental groups (Figure 2).

Finally, the model for ALT (Table 5) identified a significant effect for the interaction between measure and treatment, as well as for the main effect of sex. Specifically, between t0 and t1, the ALT value tends to increase almost significantly in the placebo group (β = 4.3 ± 2.5, t168 = 1.731, *p* = 0.085, Figure 2) and to decrease almost significantly in the treated group (β = 4.6 ± 2.5, t168 = 4.626, *p* = 0.061, Figure 2). Consequently, although at t0, the treated group exhibited a higher ALT value than the placebo group (β = 5.1 ± 2.5, t168 = 2.066, *p* = 0.040, Figure 2), this difference disappeared at measurement t1 (β = 3.7 ± 2.5, t168 = 1.490, *p* = 0.14, Figure 2). Further, regardless of size and treatment, men consistently exhibited higher ALT values than women (β = 3.6 ± 1.8, t168 = 2.041, *p* = 0.043).

## 4. Discussion

The main data presented here show that a 3-month dietary supplementation based on the intake of an ingredient of natural origin standardized to contain monacolins (4.5%), OZ, and GABA improved lipid metabolism. In particular, at the end of the food supplement treatment period (t1), the mean LDL-C and TC values showed a 19.3% and 8.3% reduction (*p* < 0.001), respectively. The mean HDL-C value increased by about 29.3% (*p* < 0.001). Since LDL-C values indicate the severity of cardiovascular risk, and that the average value of LDL-C decreases from 122 to 93 mg/dL after the treatment with the food supplement, on average, the subjects moved from very high to a moderate risk of cardiovascular disease. TC value approached the suggested normal values for this parameter (≤190 mg/dL). Although HDL-C values increase in a statistically significant manner in subjects treated with food supplements, it can be concluded that HDL-C values remain within healthy levels (>50 mg/dL). TG values were slightly decreased (*p* = 0.08) by dietary supplementation. As far as glucose metabolism is concerned, both FBG and HbA1c remained constant before and after treatment, as well as the other secondary outcomes. Finally, hepatic and renal parameters remained within normal values and no adverse events were reported to the investigator both in control and treated subjects.

Thanks to advances in diet and lifestyle, as well as improved use of antihypertensive and lipid-lowering medications, significant progress has been made in the prevention of CVD in recent decades [21,22]. The majority of dietary changes have been made in accordance with guidelines for reducing cholesterol and saturated fats [23,24,25]. Conversely, in longitudinal studies, lifetime exposure to LDL-C is highly associated with an increased risk of atherosclerotic cardiovascular disease [26,27,28]. Lipid-reducing medication is linked to a sustained decline in CV events by significantly lowering the risk. Thus, ezetimibe, PCSK9 inhibitors, and statins are proven to reduce the risk of CVDs [29]. However, gastrointestinal disorders, headaches, raised levels of liver enzymes in the serum, liver disorders, myalgia, myopathy, muscle cramps, weakness, and sleep disturbances are common adverse events related to statin use, leading to intolerance and non-adherence to therapy [30]. Hence, growing data support the use of dietary supplements together with conventional therapies to prevent CVD risk factors [31,32]. Experimental and clinical information on the safety and efficacy of food supplement ingredients acting on lipid metabolism is growing [33]. Evidence supports the use of RYR preparations among the nutraceuticals with possible lipid-lowering benefits [34,35]. The primary active ingredient in RYR, monacolin K, shares chemical similarities with lovastatin. It has been shown that RYR preparations can effectively lower LDL-C and CV events [36]. The ability of RYR to act on lipid and blood pressure lowering (and/or on inflammation) argues for a multifaceted impact of this food supplement ingredient on CV risk reduction [37].

In subjects with slightly altered cholesterol levels (TC > 200 mg/dL), supplementation of RYR extract (10 mg) for eight weeks significantly lowered (*p* < 0.001) the levels of TC by 15% and LDL-C by 22%, as compared to placebo [34]. Likewise, when compared to the placebo group, a 4-week 10 mg MK administration reduced TC (−12.45%), LDL-C (−21.99%), non-HDL-C (−14.67%), matrix metalloproteinase 2 (−28.05%), matrix metalloproteinase 9 (−27.19%), and hs-CRP (−23.77%) in 25 Italian participants with mild hypercholesterolemia [35]. In the present trial, using a significantly lower dose of MK (2.8 mg/day) in combination with OZ from rice bran (*O. sativa*) and GABA, we achieved almost similar results in terms of lipid parameters.

Our results also agree with those obtained by Minamizuka et al. [36], who studied the effect of RYR on arteriosclerosis in patients with mild dyslipidemia. Subjects in the treatment group received low-dose RYR (200 mg/day) containing 2 mg monacolin K as part of a controlled dietary therapy while subjects in the control group received dietary therapy alone for 8 weeks. Together with significant LDL-C, TC, and apolipoprotein B decreases in the RYR group compared to the control group, the treated group also showed significant decreases in blood pressure.

Moreover, during our study, no subject experienced any type of adverse event. This is in line with the results obtained by Minamizuka et al. [36], who showed that no adverse events were observed at the hepatic and renal levels in the recruited subjects and with EFSA opinion, reporting that 3 mg is the highest amount of MK to be employed in food supplement. In fact, low MK doses from RYR are expected to be associated with limited concerns about adverse events on the musculoskeletal system and on the liver in vulnerable subgroups of the population [38].

In addition to MK, RYR also contains GABA, which is a potent and inhibitory neurotransmitter of the central nervous system [39,40]. Type A GABA receptors in the brain are located at the level of the outer membranes of brain cells where the concentration of cholesterol is very high. Abnormal regulation of GABA–glutamate in the nucleus of the solitary tract is related to numerous cardiovascular comorbidity [41]. On the other hand, the intimate connection between the brain and the heart is a well-recognized event [42]. In a randomized, placebo-controlled, double-bind trial, Wang et al. [11] demonstrated the efficacy of MK-rich and GABA-rich RYR supplements in hyperlipidemia. Participants were randomized to either MK-rich RYR 250 mg (MK 8 mg/day), GABA-rich RYR 250 mg (GABA 0.14 mg/day), or placebo, twice a day for three months. Results showed a significant reduction of serum TC and LDL-C levels with MK supplementation and non-significant reduction of serum TG levels with GABA. The study suggested that using different ratios and concentrations of MK and GABA could be useful in the management of hyperlipidemia.

Preclinical studies support and extend the possibility of employing MK, OZ, and GABA (ROSSOPURO^®^ Forte-Giellepi S.p.A., Milan, Italy) in combination in clinical practice. Fifty mice were randomized in four groups, where control mice were given a standard diet (SD) for 4 weeks while the other three groups were given a high-fat diet (HFD). At the end of this period, the mice fed the SD diet continued to follow the same SD diet, while the thee groups of mice fed the HFD diet received the following treatments: one continued on the HFD diet; one continued to follow the HFD diet supplemented with a mixture of natural components derived from rice and fermented rice containing 3% monacolin K, γ-oryzanol, and γ-aminobutyric acid (HFD + S1); and the latter followed the HFD diet with the addition of monacolin K alone at 3% for 24 weeks (HFD + S2). The findings demonstrated that TC levels were lower in mice treated with HFD + S1 and HFD + S2 than in mice fed HFD alone (*p* < 0.01 and *p* < 0.05, respectively). Furthermore, TC and LDL-C levels were lower in mice treated with HFD + S1 than in animals given HFD + S2 (*p* < 0.05). TG levels were lower in mice treated with HFD + S1 or S2 than in mice fed HFD (*p* < 0.05). Thus, it may be concluded that 3% MK, OZ, and GABA together are superior to 3% monacolin K alone in mitigating the negative effects of HFD in healthy mice [10].

This study has several limitations. A possible limitation could be the absence of control over food consumption and the variation in diet composition across participants, despite the fact that the enrolled subjects were directed to follow an isocaloric diet throughout the trial. In addition, the use of a food diary and continuous questions to each volunteer about her/his adherence to the dietary plan was adopted to reduce the risk. The limited exposure to the treatment is another limitation of the present study. We plan to address this limitation in a further study, having gathered major information on the effective and safe use of ROSSOPURO^®^ FORTE in the present study. However, large comparative studies [5] argue against the possibility of major changes overtime in the efficacy and safety of dietary supplements and low-dose statins.

## 5. Conclusions

The use of food supplements in the management and prevention of CVD is supported by an increasing body of research; nonetheless, international recommendations have not given much attention to this area to date. The lack of recommendations may preclude the use of products (e.g., RYR) with potential value in particular groups of subjects (e.g., those at low CV risk). Nevertheless, vis-à-vis the lack of high-quality evidence-based recommendations for physicians and prescribers relative to the use of food factors with drug-like properties, many subjects favor self-medication with nutraceuticals and argue for ‘natural’ products being more successful and safer than traditional medicines. The current study demonstrated a significant reduction in TC and LDL-C, with a significant increase in HDL-C and slight decrease in TG values in participants with mild hypercholesterolemia, supplemented with RYR, containing a combination of MK, OZ, and GABA. In clinical practice, conventional medications with substantial evidence of reducing events should be favored; nonetheless, this study offered guidance and suggestions about the impact of food supplements on hypercholesterolemia in particular CV events (i.e., mild dyslipidemia).

## Figures and Tables

**Table 1:** Characteristics of the study population (demographic and clinical data at baseline, t0).

Characteristic	Placebo Group (*n* = 44)	Food Supplement Group (*n* = 44)
Age (years)	57 ± 7	53 ± 5
Sex:		
Females	26	20
Males	18	24
Ethnicity: Caucasian	44	44
BMI (kg/m^2^):		
Females	23.33 ± 3.56	23.30 ± 3.07
males	23.42 ± 3.84	24.08 ± 3.63
Waist circumference (cm)		
females	99.46 ± 6.15	97.85 ± 5.47
males	99.50 ± 4.59	97.66 ± 4.91

Abbreviation: BMI—body mass index.

**Table 2:** Descriptive statistics (mean, standard deviation, and range of values) for the variables measured at baseline (t0) and after 3 months of treatment (t1) with placebo or food supplement based on ROSSOPURO^®^ FORTE in the two experimental groups.

Variable	Placebo Group		Food Supplement Group	
	t0	t1	t0	t1
**FBG**	87.3 ± 10.4	87.9 ± 9.3	88.0 ± 10.2	86.8 ± 9.1
	(70–104)	(70–105)	(71–105)	(71–104)
**HbA1c**	5.0 ± 0.8	5.0 ± 0.9	5.1 ± 0.8	5.0 ± 0.9
	(4–6)	(4–6)	(4–6)	(4–6)
**TC**	217.5 ± 10.7	229.6 ± 12.7	222.7 ± 9.4	204.3 ± 11.3
	(201–239)	(210–250)	(202–238)	(184–224)
**HDL-C**	56.2 ± 14.6	51.8 ± 11.5	51.6 ± 13.1	66.7 ± 18.6
	(30–80)	(30–70)	(30–103)	(36–130)
**LDL-C**	101.6 ± 31.0	122.4 ± 27.4	115.7 ± 28.5	93.4 ± 23.8
	(56–159)	(72–163)	(57–165)	(55–140)
**TG**	98.6 ± 37.5	112.9 ± 38.0	100.4 ± 36.3	97.3 ± 25.9
	(40–165)	(62–177)	(40–163)	(42–152)
**ESR**	7.9 ± 4.2	9.1 ± 3.8	7.9 ± 3.7	8.8 ± 3.6
	(2–15)	(2–15)	(2–14)	(2–15)
**CRP**	4.3 ± 1.8	4.6 ± 1.9	4.5 ± 1.6	4.2 ± 1.7
	(2–7)	(2–7)	(2–7)	(2–7)
**WBCs**	7.5 ± 2.4	7.2 ± 2.3	7.0 ± 2.4	7.0 ± 2.4
	(4–11)	(4–11)	(4–11)	(4–11)
**AST**	19.6 ± 9.4	18.5 ± 8.9	21.5 ± 9.5	20.4 ± 8.5
	(5–35)	(6–37)	(6–37)	(6–37)
**ALT**	23.8 ± 11.7	28.2 ± 11.9	29.4 ± 11	24.8 ± 11.3
	(4–44)	(5–45)	(9–45)	(5–45)
**CRE**	1.1 ± 0.1	1.1 ± 0.1	1.1 ± 0.1	1.1 ± 0.1
	(0.91–1.28)	(0.9–1.3)	(0.9–1.3)	(0.9–1.29)

Abbreviations: FBG—fasting blood glucose; HbA1c—glycated hemoglobin; TC—total cholesterol; LDL-C—low-density lipoprotein cholesterol; HDL-C—high-density lipoprotein cholesterol; TG—triglycerides; ESR—erythrocyte sedimentation rate; CPR—C-reactive protein; WBCs—white blood cells; AST—aspartate amino transferase; ALT—alanine amino transferase; CRE—creatinine.

**Table 3:** Comparison of treatment vs. placebo: results of LMM models for the primary outcome variables.

Variable	F-Value	DF	*p*
**TC**			
Measure	3.162	1.88	0.08
Group	38.899	1.83	**<0.001**
Sex	5.169	1.82	**0.026**
Age	0.114	1.82	0.74
BMI	0.002	1.90	0.97
Waist circumference	0.955	1.99	0.33
Measure × group	85.597	1.87	**<0.001**
**HDL-C**			
Measure	5.809	1.88	**0.018**
Group	5.144	1.83	**0.026**
Sex	0.915	1.82	0.34
Age	0.174	1.83	0.68
BMI	0.146	1.90	0.70
Waist circumference	0.000	1.99	0.99
Measure × group	18.866	1.88	**<0.001**
**LDL-C**			
Measure	0.144	1.88	0.71
Group	2.059	1.83	0.16
Sex	0.006	1.82	0.94
Age	1.270	1.83	0.26
BMI	0.157	1.92	0.69
Waist circumference	2.935	1.102	0.09
Measure × group	27.617	1.88	**<0.001**
**TG**			
Measure	1.589	1.168	0.21
Group	3.865	1.168	0.051
Sex	1.261	1.168	0.26
Age	1.995	1.168	0.16
BMI	0.275	1.168	0.60
Waist circumference	3.282	1.168	0.07
Measure × group	3.166	1.168	0.08

**Table 4:** Comparison of treatment vs. placebo: results of LMM models for the secondary outcome variables.

**Variable**	**F**	**Gdl**	***p***
**FBG**			
Measure	0.097	1.88	0.76
Group	0.002	1.83	0.97
Sex	0.001	1.82	0.97
Age	0.067	1.82	0.80
BMI	0.081	1.92	0.78
Waist circumference	0.312	1.103	0.58
Measure × group	0.378	1.87	0.54
**HbA1c**			
Measure	0.129	1.87	0.72
Group	0.549	1.83	0.46
Sex	0.618	1.82	0.43
Age	0.130	1.82	0.72
BMI	0.731	1.90	0.39
Waist circumference	0.024	1.99	0.88
Measure × group	0.004	1.87	0.95
**CRP**			
Measure	0.010	1.88	0.92
Group	0.000	1.83	0.99
Sex	0.350	1.82	0.56
Age	0.080	1.82	0.78
BMI	0.037	1.91	0.85
Waist circumference	0.972	1.101	0.33
Measure × group	1.449	1.87	0.23
**WBCs**			
Measure	0.266	1.168	0.61
Group	1.072	1.168	0.30
Sex	0.317	1.168	0.57
Age	0.043	1.168	0.84
BMI	0.949	1.168	0.33
Waist circumference	0.180	1.168	0.67
Measure × group	0.296	1.168	0.59
**ESR**			
Measure	3.688	1.168	0.06
Group	0.425	1.168	0.52
Sex	0.002	1.168	0.97
Age	0.002	1.168	0.96
BMI	5.060	1.168	**0.026**
Waist circumference	1.652	1.168	0.20
Measure × group	0.201	1.168	0.65

**Table 5:** Comparison of treatment vs. placebo: results of LMM models for the secondary outcome variables regarding hepato- and nephrotoxicity.

Variable	F	Gdl	*p*
**AST**			
Measure	0.460	1.168	0.50
Group	0.913	1.168	0.34
Sex	0.961	1.168	0.33
Age	0.012	1.168	0.91
BMI	0.003	1.168	0.96
Waist circumference	0.981	1.168	0.32
Measure × group	0.000	1.168	0.98
**ALT**			
Measure	0.010	1.168	0.92
Group	0.151	1.168	0.70
Sex	4.165	1.168	**0.043**
Age	0.085	1.168	0.77
BMI	0.012	1.168	0.91
Waist circumference	0.165	1.168	0.68
Measure × group	6.546	1.168	**0.011**
**CRE**			
Measure	0.104	1.168	0.75
Group	0.810	1.168	0.37
Sex	0.599	1.168	0.44
Age	0.270	1.168	0.60
BMI	0.138	1.168	0.71
Waist circumference	0.862	1.168	0.35
Measure × group	1.377	1.168	0.24

## Data Availability

The original contributions presented in this study are included in the article; further inquiries can be directed to the corresponding authors.
